# Detecting CO_2_-Sensitive Hemichannels in Neurons in Acute Brain Slices

**DOI:** 10.1016/j.xpro.2020.100139

**Published:** 2020-10-23

**Authors:** Emily Hill, Nicholas Dale, Mark J. Wall

**Affiliations:** 1 School of Life Sciences, University of Warwick, Coventry CV4 9YH, UK

## Abstract

This protocol provides two independent methods to functionally detect the neuronal expression of CO_2_-sensitive hemichannels. These hemichannels (consisting of connexins 26 or 30) are directly gated by CO_2_, independent of pH changes and until recently were thought to be only expressed by glia. This protocol outlines a method to change the concentration of CO_2_ without changing pH, using isohydric solutions and then utilizing this to detect opening and closing of functional hemichannels using whole-cell patch clamp recording and dye loading.

For complete details on the use and execution of this protocol, please refer to Hill et al. (2020).

## Highlights

• Protocols for detecting CO_2_-sensitive hemichannels in neurons of acute brain slices
• Use of electrophysiology to look for changes in neuronal firing and input resistance
• Use of dye loading to confirm neuronal CO_2_-sensitive hemichannel expression
• Use of immunohistochemistry to confirm connexin expression in neurons of interest

## Graphical Abstract

**Figure:**
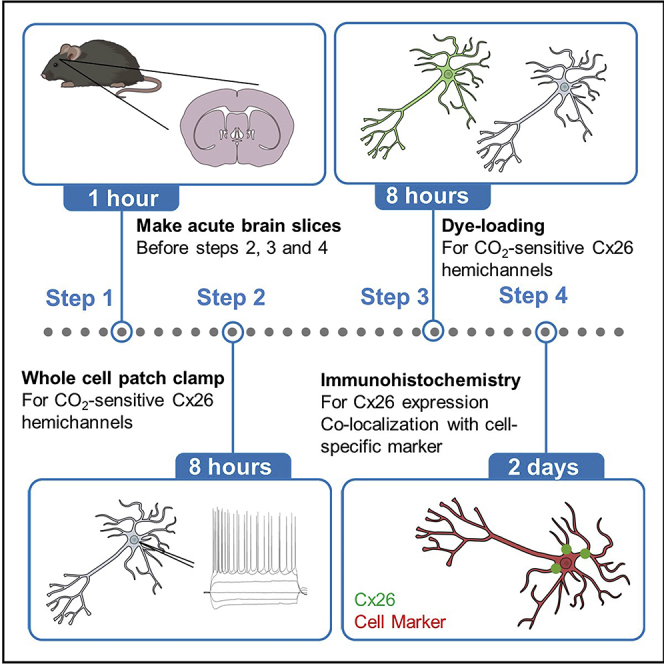

## Before You Begin

***Note:*** We use the following abbreviations in this protocol:

**Table:** 

Cx26	Connexin 26
SIV	Standard IV (I- current; V- voltage)
DIV	Dynamic IV (I- current; V- voltage)
CBF	Carboxyfluorescein
IHC	Immunohistochemistry

### Electrophysiology Setup

**Timing: 1.0–1.5 h**

1. The researcher will need access to a whole-cell patch clamp electrophysiology recording rig to be able to record from the neurons of interest in acute brain slices. For the dye-loading experiments, there is an additional requirement for fluorescent illumination which could be part of a patch clamp or calcium imaging rig. The fluorescence needs to be ~488 nm with a total magnification of at least x400 (x40 objective and x10 camera magnification) with the facility to record images.
2. Acute brain slices containing the relevant brain area (recommendation of 300–350 μm thickness) first have to be prepared and can then be stored at 34 ˚C in 35 mmHg CO_2_ buffer (see recipes below). Slices can be stored for up to 6–8 h post-slicing.

***Note:*** Standard slice preparation protocols will generate brain slices that can be stored and used between 1 to 8 h post slicing. This time will differ depending on the age of the animal and the region of interest. Always allow the slices a minimum of 1 hour for recovery after slicing before experiments.

***Note:*** This protocol can be applied to both mouse and rat brain slices. It can be used with any region of interest and any age of animal as required by your study. We have tested dopaminergic neurons in the substantia nigra pars compacta and GABAergic neurons in the ventral tegmental area using two different ages of mice (P7–10 and P17–21) and showed CO_2_ sensitivity. As a control, we also demonstrated that there was no CO_2_ sensitivity in the CA1 region of the hippocampus using both dye loading and electrophysiology. See Hill et al (2020) for more details.

3. The researcher will need a 95% O_2_ and 5% CO_2_ supply, an 100% CO_2_ supply, and an 100% O_2_ supply. The researcher will need mixer valves so the gases can be mixed to produce the correct levels of CO_2_ for the experiments (see below for details) and a pH meter and electrode.

**CRITICAL:** It is vital to correctly use the mixer valves to ensure a constant pH across the two solutions (20 or 55 mmHg CO_2_, relative to 35 mm Hg) as this protocol requires isohydric (constant pH) conditions in order to isolate pH-independent CO_2_-mediated effects. Always monitor the pH of the solutions throughout the day, to ensure that the pH does not drift over time. Although the mixer valves take more time and monitoring than pre-bought gases, they allow for much greater experimental flexibility so that bespoke mixtures can be created as required.

***Note:*** The midpoint for connexin 26 hemichannel opening is around 35 mmHg CO_2_. Therefore, if cells are sensitive, then fluctuations above this (to 50 mmHg CO_2_) and below this (to 20 mmHg CO_2_) would be expected to alter the open probability of the channels and thus the cells would display a conductance phenotype.

***Note:*** For the electrophysiology, we carry out two different experiments: firstly, we change from 35 to 55 mmHg CO_2_ buffer to increase the opening of the hemichannels and in separate experiments we change from 35 to 20 mmHg CO_2_ buffer to increase the closing of the hemichannels.

### Isohydric Solution Preparation

**Timing: 30 min**

***Note:*** The baseline solution (buffer) is always 35 mmHg CO_2_. Depending on experiment, either 55 mmHg or 20 mmHg CO_2_ buffer will also need to be prepared. These buffers differ in their concentrations of NaCl and NaHCO_3_ (see Materials and Equipment for further details).

**Figure 1:**
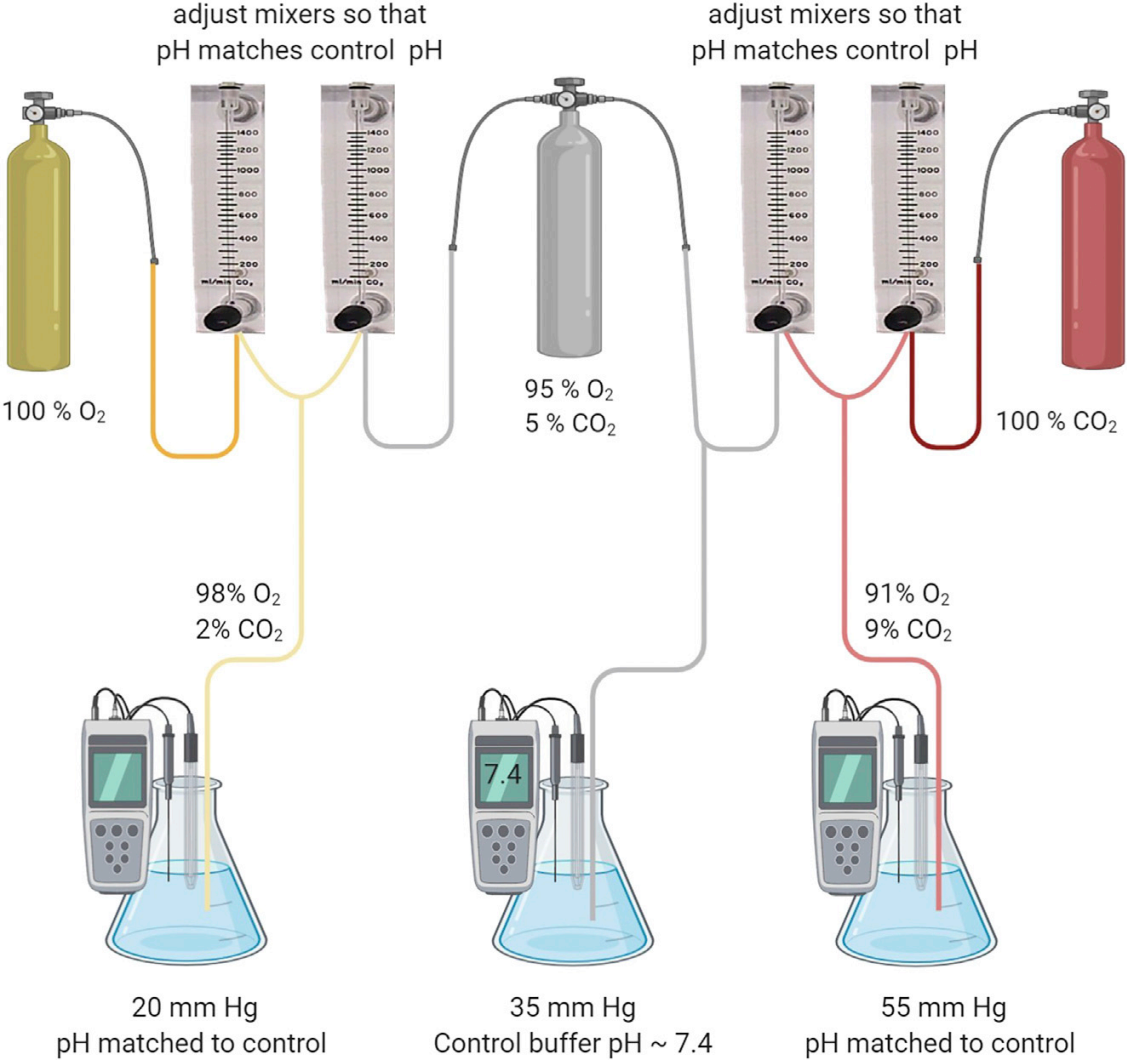
Mixer Setup to Enable Smooth Isohydric CO_2_ Level Change The 35 mmHg CO_2_ buffer is bubbled with 95% O_2_/5% CO_2_. The pH of this buffer should not need to be altered and should be ~7.4. The 55 mmHg CO_2_ buffer is saturated with 9% CO_2_ (with the balance being O_2_) with pH maintained to match control (35 mmHg CO_2_). This is achieved by mixing the 95% O_2_/5% CO_2_ line with a 100% CO_2_ line (adjustments are made using the mixer dials). The 20 mmHg CO_2_ buffer is saturated with 2% CO_2_ (with the balance being O_2_), with pH maintained to match control (35 mmHg CO_2_). This is achieved by mixing the 95% O_2_/5% CO_2_ line with a 100% O_2_ line (adjustments are made using the mixer dials). Created with BioRender.com

***Note:*** Before starting, ensure that you have the mixers set up correctly (see Figure 1). There are a number of suppliers for mixers, we use PLATON Variable-area flow meters. If you would rather buy bespoke pre-made CO_2_/O_2_ mixtures to avoid having to mix your own, you can do this at an extra cost.

***Note:*** For 20 mmHg CO_2_ buffer you will need 2 × 0–10 mL/MIN AIR × 100 (6831), one for the 100% O_2_ line and one for the 95% O_2_/5% CO_2_ line to mix the two gases in a roughly 1:1 proportion.

***Note:*** For 55 mmHg CO_2_ buffer, you will need 1 × 0–250 mL/MIN AIR × 100 (6831) for the 95% O_2_/5% CO_2_ line and 1 × 0–10 mL/min AIR (6387) for the 100% CO_2_ line, to mix the gases in a roughly 0.96 to 0.04 proportion.

***Note:*** All buffers should have an osmolarity of 300 mOsm. This can be a good step for troubleshooting.

4. Generation of isohydric solutions to vary CO_2_ levels without changing the extracellular pH.
  a. Prepare the 35 mmHg CO_2_ buffer and either the 55 mmHg or 20 mmHg CO_2_ buffer depending on whether you want to open or close the hemichannels. Use the table in Materials and Equipment for the buffer recipes (solutions based on Huckstepp et al (2010))
5. Generating correct gas mixtures and correcting for pH differences
  a. The 35 mmHg CO_2_ buffer is bubbled with standard 95% O_2_/5% CO_2._ The pH for this solution does not need to be altered but should be checked each day to ensure that it is ~7.4. Test this first before beginning the mixer preparation for (b) and (c). If the solution is not ~pH 7.4, then remake to ensure no errors have been made.
  b. The 55 mmHg CO_2_ buffer is saturated with 9% CO_2_ (with the balance being O_2_) with pH matched to control (35 mmHg CO_2_ buffer). This is achieved by mixing the 95% O_2_/5% CO_2_ with the 100% CO_2_ using the mixer valves.
    i. Place the pH electrode into the 55 mmHg CO_2_ buffer solution. Make small adjustments to the levels of the 95% O_2_ 5% CO_2_ and 100% CO_2_ by turning the black dials at the bottom of the mixer. Allow 5–10 min for equilibration and then read the pH from the meter.
    ii. Continue this process until the pH matches that of the 35 mmHg CO_2_ buffer solution.
    iii. Periodically check throughout the day that the pH has not altered.
  c. The 20 mmHg CO_2_ buffer is saturated with 2% CO_2_ (with the balance being O_2_), with pH matched to control (35 mmHg CO_2_ buffer). This is achieved by mixing the 95% O_2_ 5% CO_2_ with 100% O_2_ using one of the mixer valves.
    i. Place the pH electrode into the 20 mmHg buffer solution. Make small adjustments to the levels of the 95% O_2_ 5% CO_2_ and 100% O_2_ by turning the black dials at the bottom of the mixer. Allow 5–10 min for equilibration and then read the pH from the meter.
    ii. Continue this process until the pH matches that of the 35 mmHg CO_2_ buffer solution.
    iii. Periodically check throughout the day that the pH has not altered.

***Note:*** Setting up and calibrating the mixers will take some time on the first day of experiments, but if the mixer dials are left at the end of the experiments, then only minor adjustments will be needed for subsequent experiments.

### Preparation of Solutions for Dye Loading Experiments

**Timing: 10 min**

6. Generate isohydric solutions as outlined above (35 mmHg CO_2_ and 55 mmHg CO_2_).
  a. To 200 mL of the 35 mmHg CO_2_ buffer and 55 mmHg CO_2_ buffer add 15 mg of the dye carboxyfluorescein and mix thoroughly.
  b. Bubble as normal in the water bath until use

## Key Resources Table

**Table:** 

REAGENT or RESOURCE	SOURCE	IDENTIFIER
**Antibodies**		

Mouse monoclonal anti-Connexin 26	Invitrogen	138100
Donkey anti-mouse 594	Invitrogen	A21203

**Chemicals, Peptides, and Recombinant Proteins**		

Carbenoxolone disodium salt	Sigma Aldrich	C4790-1G
(6)-Carboxy-fluorescein (CBF)	Novabiochem	8.51082.001
NaCl	Fisher	S/3160/65
NaHCO_3_	Alfa Aesar	A17005
NaH_2_Po_4_	Fisher	P/4800/53
KCl	Fisher	P/4280/53
D-Glucose	Fisher	G/0500/53
MgSO_4_	Fisher	M/1050/53
CaCl_2_ (1 mM stock)	In-house	NA
Triton X-100	Fisher	T/3751/08
BSA	G-Biosciences	224B-B
Vectasheild	Vector Labs	H-1000
HEPES	Sigma	H4034
Potassium D-Gluconate	Sigma	G4500
EGTA	Sigma	E3889
MgATP	Sigma	A9187
GTP	Sigma	G9002
Phosphocreatine	Sigma	P7936

**Software and Algorithms**		

pClamp suite (version 10)	http://www.moleculardevices.com/products/software/pclamp.html	RRID:SCR_011323
IC Capture Version 2.4.642.2631	Imaging source	NA
Zen Black	http://www.zeiss.com/microscopy/en_us/products/microscope-software/zen.html#introduction	RRID:SCR_013672

**Experimental Models: Organisms/Strains**		

C57/BLK6 wild-type mice 2-3 weeks old	Bred in-house Other strains can be used as required	NA

**Other**		

Microm microslicer	Thermoscientific	HM 650V
Gibbs slice Preincubation chamber	In-house (Warner supply a version)	64-125/65-0076
Anti-vibration table	TMC vibration control	Clean bench (cat depends on size)
Faraday cage	In-house (TMC supply a version)	TMC 81-334∗
Temperature controller	Scientifica in-line Peltier heater	SM-4600∗
Fixed-stage upright microscope with infrared and fluorescence filters	Scientifica	Olympus BX151W or SliceScope∗
Fluorescence microscopy illumination system	CoolLED	PE-4000-L-SYS∗
CCD camera	Hitachi	KP-F1AP∗
Motorized Manipulators	Scientifica	PatchStar or MicroStar∗
Patch clamp amplifier	Molecular Devices, USA	Axon Multiclamp 700B∗
Low-noise digitizer	Molecular Devices, USA	Digidata 1440A∗
Perfusion pump	Watson Marlow	120S/DV∗
Water baths ×2	Clifton	FL28D∗
100% O_2_ Line	BOC Medical oxygen	PL0735/5000∗
100% CO_2_ Line	In-house	NA
95% O_2_/5% CO_2_ line	In-house	NA
Microelectrode puller	Sutter	P97
Patch pipettes (5–10 MΩ resistance)	Manufactured from thick walled glass (Multichannel systems)	300057
PLATON Variable-area flow meters	2 × 0–10 mL/MIN AIR × 100 and 1 × 1 × 0–10 mL/min AIR Originally purchased from Roxspur	6831 6837

∗There are many good options for electrophysiology rig components. Here we have listed the components of our rig, but this can be altered to any standardized patch clamp rig.

## Materials and Equipment

**Table:** Solution recipes for isohydric buffers

	35 mmHg CO_2_ buffer (control)		55 mmHg CO_2_ buffer (hypercapnic)		20 mM Hg CO_2_ buffer (hypocapnic)	
Reagent	Final Conc (mM)	Amount (g)	Final Conc (mM)	Amount (g)	Final Conc (mM)	Amount (g)
NaCl	124	7.2466	100	5.8440	140	8.1816
NaHCO_3_	26	2.1843	50	4.2001	10	0.8401
NaH_2_Po_4_	1.25	0.1950	1.25	0.1950	1.25	0.1950
KCl	3	0.2237	3	0.2237	3	0.2237
Glucose	10	1.8016	10	1.8016	10	1.8016
MgSO_4_	1	0.2465	1	0.2465	1	0.2465
CaCl_2_ (1 mM stock)	2	2 mL	2	2 mL	2	2 mL
dH_2_0	n/a	**To 500 mL**	n/a	**To 500 mL**	n/a	**To 500 mL**
Bubbled with:	*95% O*_*2*_*5% CO*_*2*_*with a final pH of ~7.4*		*9% CO*_*2*_*(with the balance being O*_*2*_*) with pH maintained to match control (35 mmHg)*		2% CO_2_ (with the balance being O_2_), with pH maintained to match control (35 mmHg)

***Note:*** A 10× stock can be made including NaCl, NaHCO_3_, NaH_2_PO_4_ and KCl and stored at 4 ˚C until the day of use. Glucose and CaCl_2_ must be added on the morning of each experiment.

***Note:*** The buffers were originally confirmed to be either 35, 20, or 55 mmHg CO_2_ using a blood gas analyzer. Each of the solutions was tested in triplicate. They have since been used routinely in the lab. If you wanted to use a new level of CO_2_, then you would need to alter the buffer recipe accordingly and measure the level of CO_2_ (mmHg) with an appropriate method (such as gas analyzer or CO_2_ electrode). We found that the composition of the buffer required to give the desired pCO_2_ cannot be predicted using the Henderson Hasselbalch equation, which we think is due to the presence of other ions in the buffers.

**CRITICAL:** When making the solutions, add all reagents except CaCl_2_ and 50 mL of dH_2_0. Bubble this solution for 10 min with 95% O_2_/5% CO_2_, then add CaCl_2_, and top up with the remaining dH_2_0 to avoid any precipitation.

## Step-By-Step Method Details

### Electrophysiological Detection of Hemichannels in Neurons in Acute Brain Slices

**Timing: 5–6 h**

This protocol provides a method for testing using electrophysiology whether changes in CO_2_ levels can modulate neuronal excitability.

**Figure 2:**
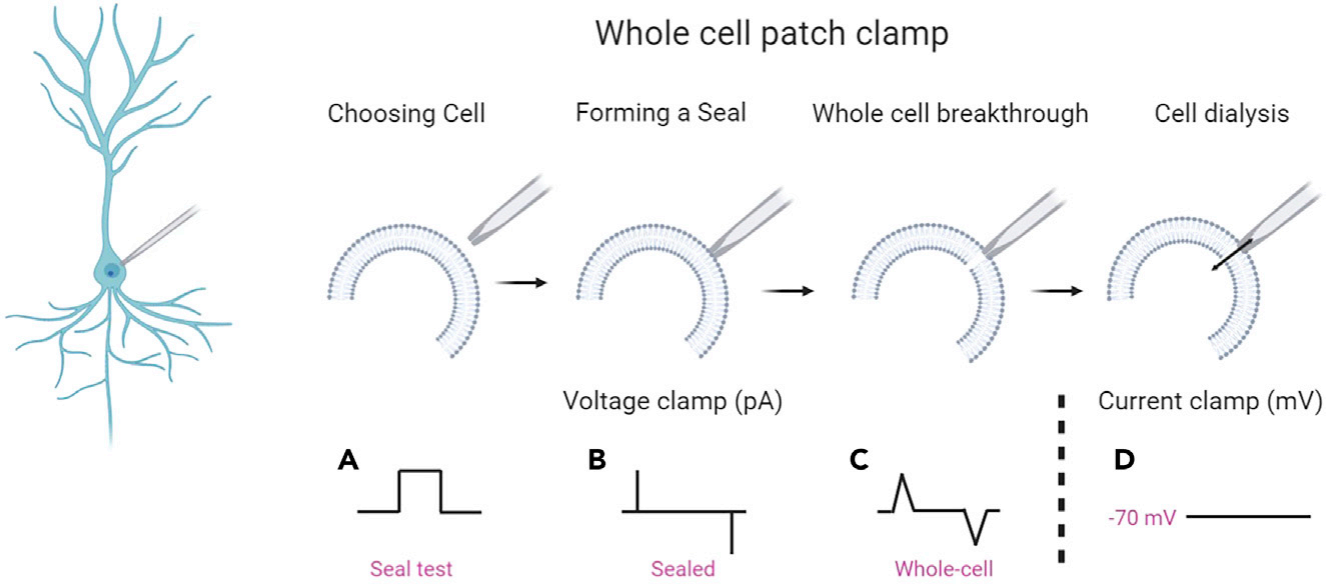
Simplified Schematic of the Whole-Cell Patch Clamp Protocol Firstly, identify the neuron of interest using the brightfield image. Then using a glass pipette (5–7 MΩ resistance) approach the cell with positive pressure on the pipette. We monitor and control the pressure with our mouths, but it is also possible to use a syringe to apply pressure (but there is less control). Upon making contact with the target cell you should see a dimple form on the cell at the end of the pipette (as a result of the positive pressure). To monitor the resistance of the pipette and sealing, a square step voltage of 5-10 mV (at 1 Hz, as shown in A) is applied. The amplitude of the current response gives a measure of the resistance B) Once the dimple is observed, release the pressure and this will allow a high resistance seal to form between the end of the pipette and the cell membrane (some negative pressure may also need to be applied). Since I = V/R (Ohms law), as the resistance of the pipette goes up (as the pipette seals to the membrane), the current response will get smaller until the trace appears flat with only the pipette capacitance transients remaining (B). The seal resistance needs to be at least 1 GΩ but hopefully higher for a good recording (if the seal is poor then a new recording has to be made from a new cell, using a new pipette). The pipette transients can be zeroed off at this point. Increasing negative pressure is slowly applied to the pipette in order to rupture the cell membrane under the pipette and this allows break through into whole-cell. At this point the capacitance transients for the cell are detected (C). At this stage we would then move from voltage clamp into current clamp mode (the resting potential for a good recording will be ~−60 to −70 mV depending on cell type) (D). The contents of the patch pipette will start to dialyze into the cell and it is good to wait for 5–10 min for equilibration before starting recording. See Figure 3 for suggested recording protocols. Created in part with BioRender.com

**Figure 3:**
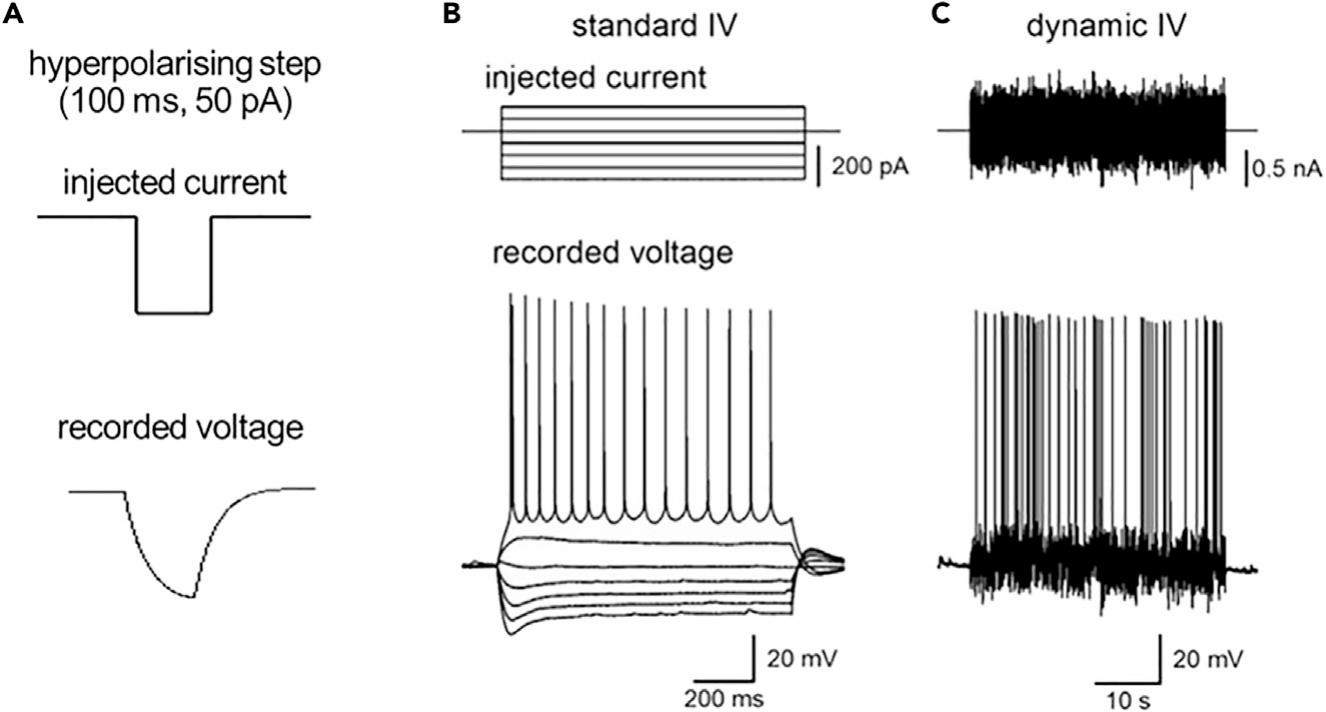
Suggested Stimulation Protocols In our experiments we run three different current input protocols in order to fully assess the effects of the CO_2_ sensitivity on neural function. (A) The first is a repeated hyperpolarizing step of −50 pA, for 100 ms, delivered every second. This is given continuously and can therefore provide a measure of the voltage response and input resistance over time. (B) The second is a standard stepwise current voltage (IV) protocol, often referred to as a SIV protocol. The current steps to use and the voltage response will depend on the cell type that you are recording from. Here is an example from a CA1 hippocampal pyramidal neuron, where current steps from −300 pA increasing by 50 pA have been introduced. The protocol should be run until a regular firing pattern is induced. This can be used to assess cell identity and health and to measure resting membrane potential and input resistance (from the current steps around the membrane potential). (C) The third current injection protocol (as used to produce a dynamic IV) injects a naturalistic current into the cell (top panel) and the voltage recorded (bottom panel) can be used to extract to determine the firing rate under a naturalistic state (for more detail, see Badel et al, 2008, Harrison et al, 2015; used in the DIV protocol). Figure reproduced in part using content previously published in Hill et al. (2019); eNeuro on a CC-BY 4.0 license.
    ii. Begin running current steps (50 pA hyperpolarizing step, 1s interval; Figure 3A) so you can monitor the input resistance of recorded neuron.
    iii. Generate a baseline of current steps over 10–15 min.

1. Making recordings from identified neurons in acute brain slices
  a. Perfuse the recording bath (2–3 mL/min) with the 35 mmHg CO_2_ buffer (bubbled with 95% O_2_ /5% CO_2_) and allow to reach recording temperature (~30 ˚C).
  b. Place brain slice in the bath (hold in place using a slice hold down for example, Multichannel systems #64-1418) and allow to equilibrate for 10–15 min
  c. Make patch clamp recording in current clamp mode from neuron(s) of interest. See Figure 2 for details of the process or refer to the axon guide (Molecular Devices, 2020) for a more thorough overview of the patch clamp technique.
    ***Note:*** Any standard patch clamp electrophysiology rig can be used for this protocol. There are many possible options for rig set up, but for clarity our slices were visualized using IR-DIC optics with an Olympus BX151W microscope (Scientifica, Bedford UK) and a CCD camera (Hitachi). Voltage recordings were made using an Axon Multiclamp 700B amplifier (Molecular Devices, USA) and digitized at 20 KHz (Digidata 1440A; Molecular devices, USA). Data acquisition and analysis were performed using pClamp 10 (Molecular Devices, USA).
    ***Note:*** Patch pipettes (5–10 MΩ) were manufactured from thick walled glass (Multichannel systems).
    ***Note:*** Use any standard intracellular patch solution for making recordings. We use and would recommend the following composition: 135 mM potassium gluconate, 7 mM NaCl, 10 mM HEPES, 0.5 mM EGTA, 10 mM phosphocreatine, 2 mM MgATP, and 0.3 mM NaGTP (293 mOsm, pH 7.2).
    ***Note:*** Always ensure intracellular solution is filtered directly before use.
    i. Carry out the standard IV protocol (Figure 3B) to check the health of the neuron, seal stability and identity (resting membrane potential, firing pattern) and allow cell to equilibrate for 5 min. Can also inject naturalistic current to measure firing rate (Figure 3C).
      **CRITICAL:** Monitor the bridge balance/ series resistance across recordings to ensure stability. Any recordings where this diverges by more than 20% over the period of recording should be disregarded.

2. Changing the level of CO_2_ to open or close hemichannels
  a. Change perfusion of the slice from 35 mmHg CO_2_ buffer to either 55 mmHg CO_2_ buffer (to open hemichannels) or to 20 mmHg CO_2_ buffer (to close hemichannels)
    i. Continue running current steps for at least 10–15 min
    ii. Once stable, carry out standard IV and inject naturistic current injections to measure firing rate.
  b. ***Optional:*** Change perfusion of slice back to 35 mmHg CO_2_ buffer to attempt reversal of the effect. Note that this can be quite challenging.
    i. Run current steps for a further 10–15 min

***Note:*** Ensure that you save all SIV, firing rate traces, and sweep files of the current steps for analysis. A measure of the size of the voltage response can be taken from each step. Steps can be averaged easily in time-bins if required in Clampfit. SIV measurements can be used to measure resting membrane potential and input resistance and the voltage response to the naturalistic current injection can give a measure of the cells firing.

***Note:*** In our study we chose to record in current clamp (recording the cell’s membrane potential) as this allowed us to run the SIV protocol to validate cell type at the start of recording and also inject naturalistic current to look at firing. This protocol could also be used to record the current directly (in voltage clamp), although additional stimulation protocols will need to be used.

### Dye Loading of Neurons through Hemichannel Opening

**Timing: 5–6 h**

**Figure 4:**
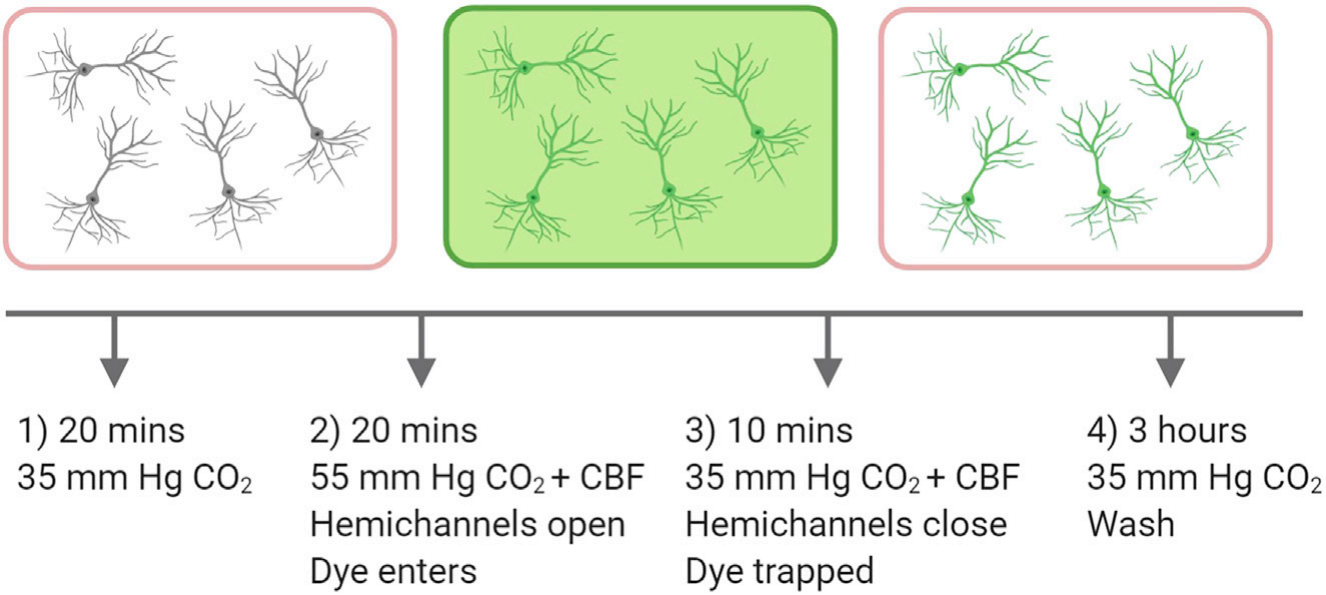
Simplified Schematic of the Dye Loading Protocol A slice is transferred to the recording chamber, submerged, and perfused with control aCSF (35 mmHg CO_2_) at 30°C. Whole-cell patch clamp recordings can be used to confirm the identity of the target region of cells. Slices were then allowed to equilibrate for 20 min. The control aCSF was then exchanged for 55 mmHg CO_2_ aCSF (hypercapnic) containing 5(6)-carboxy-fluorescein (CBF, 100 μM) for 20 min to allow the CO_2_ sensitive-hemichannels to open. The solution was then exchanged for 35 mmHg CO_2_ aCSF containing CBF (100 μM) for 5 min to allow the hemichannels to close. Finally, the slice was washed with 35 mmHg CO_2_ aCSF for 3 h to reduce the background staining before imaging. The dye loading method is based on that described in Huckstepp et al (2010). Created with BioRender.com

This protocol outlines the fluorescent imaging of neurons that express CO_2_ sensitive hemichannels by trapping a cell impermeant dye (which can move through open hemichannels). The protocol is simplified in Figure 4.

3. Preparing a slice for the dye loading of neurons
  a. Perfuse recording bath with the 35 mmHg CO_2_ (control) buffer (bubbled with 95% O_2_ /5% CO_2_) and allow to reach recording temperature.
  b. Place brain slice in the bath and allow to equilibrate for at least 15 min. Researcher could make initial recordings from neurons or use landmarks to ensure that they are imaging the correct region of the slice.

***Note:*** If performing dye-loading experiments following electrophysiology, you could potentially include in your pipette solution a different membrane impermeable dye so that you could identify with dye loading the neurons you recorded from and found to be sensitive to CO_2_ changes.

4. Opening hemichannels
  a. The control buffer is exchanged for 55 mmHg CO_2_ aCSF buffer (hypercapnic) containing 5(6)-carboxy-fluorescein (CBF, 100 μM) for 20 min to allow the CO_2_-sensitive hemichannels to open.
5. Closing hemichannels and trapping dye
  a. Solution is exchanged for 35 mmHg CO_2_ buffer containing CBF (100 μM) for 5 min to allow the hemichannels to close, trapping the dye inside.
  b. Solution is exchanged to 35 mmHg CO_2_ aCSF without the dye for at least for 3 h to reduce the background staining before imaging.
6. Imaging of filled cells
  a. Images are taken using a CCD camera with 488 nm fluorescence (CoolLED). As CBF rapidly bleaches, images need to be quickly acquired from regions of interest. CBF cannot be fixed using PFA (as it lacks the required groups for cross-linking).

**CRITICAL:** Do all the dye loading without the microscope light on to avoid bleaching. Prolonged washing of the dye is required for brain slices compared to use of cultured cells or cell lines due to the thickness of the tissue. The dye rapidly bleaches so it is important to take the images rapidly.

***Note:*** A suitable control is to not change the buffer from 35 mmHg CO_2_ and show neurons do not dye load. It is also possible to block dye loading with carbenoxolone (100 μM; an indiscriminate hemichannel blocker) to further show that the effects are hemichannel mediated.

***Note:*** We use CBF as we know it passes through connexin 26 hemichannels. It is not fixable with PFA as it lacks the required groups for cross-linking. There may be other dyes that can pass through the hemichannels and can be fixed. A trial and error approach would have to be used to discover such dyes.

### Immunohistochemistry to Confirm Connexin Expression Profile

**Figure 5:**
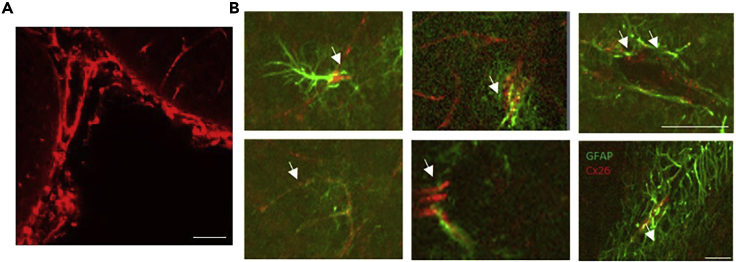
Representative Examples of Co-localization Imaging for Connexin 26 with a Cell Group of Interest (A) A positive control for successful connexin 26 staining (leptomeninges, red). (B) Co-localization staining of connexin 26 (red; 594) with glial cells (GFAP; green; 488). Images were acquired on a Leica 880 confocal and processed using Zen Black software following the above immunohistochemistry protocol detailed above. Scale bars, 50 μm. Figure reproduced in part using content previously published in Hill et al. (2020); iScience on a CC-BY 4.0 license.

This protocol outlines how to confirm the connexin expression profile of your region of interest, as further evidence of the observed effects being mediated by CO_2_-sensitive hemichannels (in this case Cx26, see Figure 5).

***Note:*** For the best results with immunohistochemistry, mice/ rats should be cardiac perfused 4% PFA and then post-fixed overnight at 4 ˚C (18–24 h).

7. The slices are then washed 5 times in PBS.
8. They are then blocked for 1 h (1% BSA, 0.4% Triton X-100 in PBS, 400 μL per slice).
9. The slices are washed 5 times in PBS.
10. The primary antibodies: a marker for your cell of interest (at required concentration; Green 488) and an antibody against connexin 26 (1:200, Mouse; Red 594) are added to the slices (400 μL per slice) for 1 h at room temperature (20°C–22°C) on a shaker and then kept at 4°C–8°C overnight (12–16 h) static in the fridge.
11. Slices are washed five times for 5 min with PBS.
12. The corresponding secondary antibodies are then added (400 μL per slice) for 4 h at room temperature (20°C–22°C).

***Note:*** Cover the plate in foil after addition of the secondary antibodies to avoid contact with light. Maintain all further steps in as little light as possible.

13. The slices are washed 5 times with PBS
14. Slices are mounted on glass slides with Vectashield (Vector laboratories, Peterborough UK) and stored in foil at 4 ˚C until imaging.

***Note:*** The earlier the imaging can be done following mounting the better. All our imaging was completed within 2 weeks of the initial immunohistochemistry to ensure the best staining.

***Note:*** Any standardized protocol for imaging can be used. The immunohistochemistry protocol will need to be optimized for the antibodies that you are using and the cells of interest. We image with a Leica 880 confocal microscope and perform image acquisition and processing in the Zen black software suite.

## Expected Outcomes

This protocol describes a way in which you can look for a CO_2_-sensitivity phenotype among a specific group of neurons of interest across different areas of the brain. Electrophysiology provides the first evidence of a neuronal response to changing the level of CO_2_ and as the solutions are kept at a constant pH (isohydric), the changes are completely independent of pH. Connexin 26 and 30 are known to be directly CO_2_ sensitive and open in response to raised levels of CO_2_ (Huckstepp et al, 2010; Meigh et al, 2013). If they are expressed and form functional hemichannels in the cells of interest, then an increase of the CO_2_ level would be expected to increase the opening probability of hemichannels and thus cause an increase in conductance (decrease in input resistance), which can be measured by whole-cell patch clamp. The firing rate of the neuron is also a good measure of change, as a drop in input resistance should lead to a decrease in excitability.

**Figure 6:**
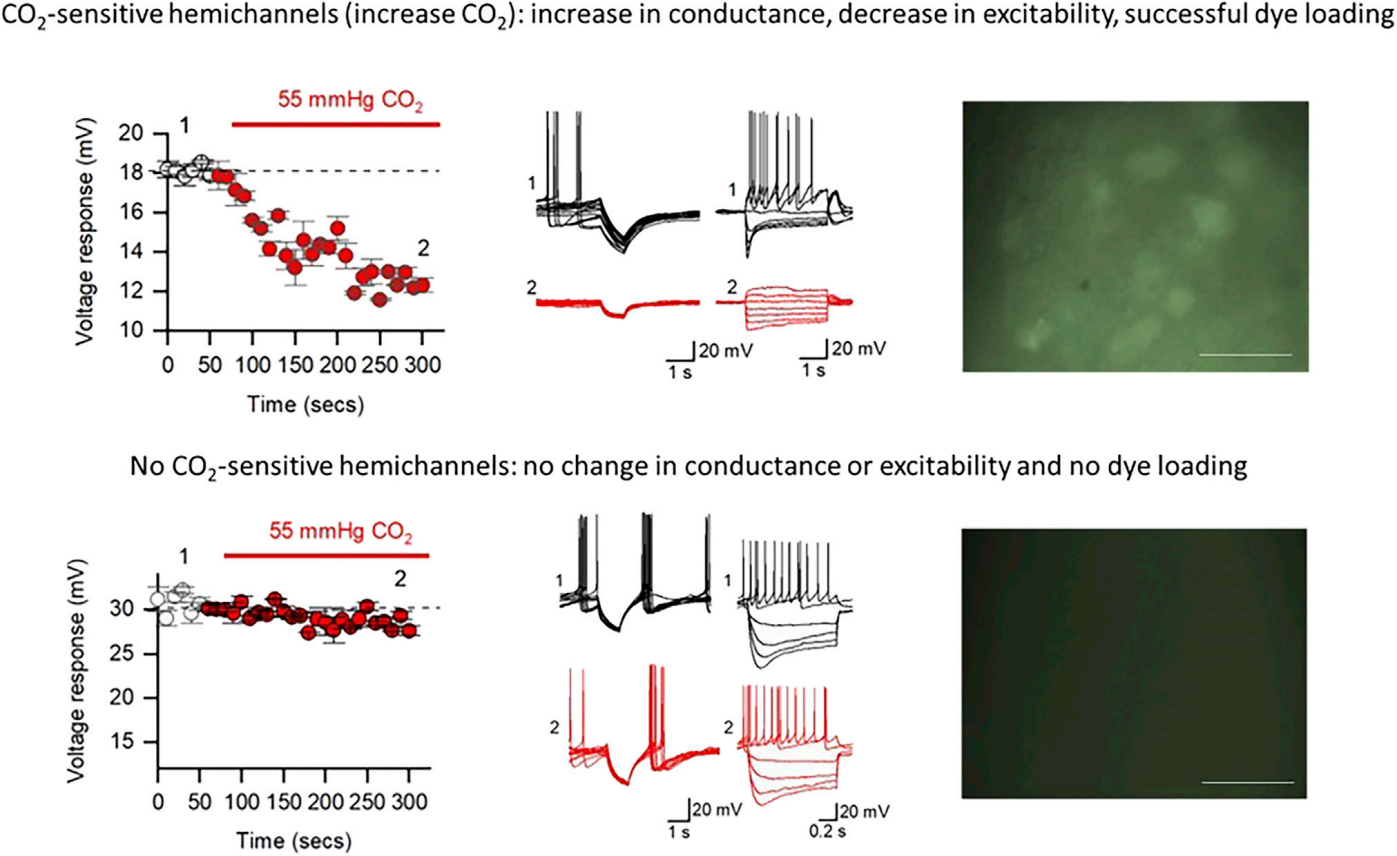
Expected Outcomes for Electrophysiology and Dye-Loading Experiments The top row represents results from a group of neurons that do express Cx26 as functional hemichannels. (Left) Voltage time course when switching from 35 mmHg CO_2_ to 55 mmHg CO_2_ buffer. A reduction in amplitude can be observed which is characteristic of a decrease in input resistance (increase in conductance, suggesting the opening of a membrane channel). (Middle) Step and SIV responses in 35 (black) and 55 (red) mmHg CO_2_ from a representative cell, displaying the decrease in input resistance and excitability upon hemichannel opening. (Right) Successful dye loading will appear as green filled neurons. The bottom row represents results from a group of neurons that do not express Cx26 as functional hemichannels. Here, there is no change to conductance or excitability and no successful dye loading. Confirming that there is no presence of functional CO_2_-sensitive hemichannels in these neurons. Reproduced in part using content previously published in Hill et al. (2020); iScience on a CC-BY 4.0 license. Scale bars, 50 μm. Data are presented as means ± SEM.

Figure 6 outlines what the researcher would observe in two cases: firstly, where CO_2_ sensitive hemichannels are present (Figure 6; top) and secondly where CO_2_ sensitive hemichannels are not present (Figure 6; bottom). In the case of presence, there is a steady reduction in the size of the response to a 50 pA (100 ms) hyperpolarizing current step over time. The full time course can be seen on the left and examples before and after raised CO_2_ of the voltage response to the step and SIV injection in the middle. This tells you that the neurons are responding to CO_2_. In the case of no CO_2_ sensitive hemichannels present, there is no significant change to conductance or excitability over time.

***Note:*** Always perform the relevant controls, suggestions of appropriate controls are detailed next. First, repeat the experiments but with no change in the level of CO_2_ (just by switching solutions) to ensure it is not an artifact of this. Secondly, repeat the experiments with carbenoxolone (100 μM; an indiscriminate hemichannel blocker), if the effect is real and through hemichannels then it will be abolished in this case. Finally, as a control try a different unrelated region of the brain (that has not previously been shown to be CO_2_-insensitive). In our study we used the CA1 region of the hippocampus and showed no electrophysiological phenotype or dye loading.

Dye-loading experiments can then take you a step further to confirming that the changes are mediated by connexin 26. Here, you will use (6)-Carboxy-fluorescein (CBF), as it is known to dye load through connexin 26 in raised CO_2_. If the cells in your field of view have functional hemichannels then they will load with CBF (Figure 6; top right). If they do not, then no dye loading will be observed (Figure 6; bottom left).

Finally, immunohistochemistry can be performed to look for co-localization between the expression of CO_2_ sensitive connexins (26 and 30) with your neuron / cell of interest by looking for co-localization.

## Quantification and Statistical Analysis

All data processing and analysis can be performed in Clampfit which is available with the pClamp suite of software. All imaging was carried with confocal microscopy (Leica 710 and Zen Black for image acquisition and processing). Appropriate statistical tests were chosen based on sample size, whether there were repeated measures and whether the populations were paired or unpaired (Wilcoxon rank sum and Mann Whitney tests respectively). For tests of more than two variables, Kruskal-Wallis ANOVAs were run with Dunn’s post hoc multiple comparisons. All tests were run to find significance at the level p < 0.05 and were performed on raw (non-normalized) data.

## Limitations

This protocol provides a way to test for the presence of functional (pH independent) CO_2_-sensitive hemichannels in different cell populations. The combination of electrophysiology with dye loading and immunohistochemistry can provide you with a strong foundation of understanding of expression and functional consequence of CO_2_-sensitivity within your cells. There are three main limitations to the study, the first is that it does not provide a behavioral correlate to the sensitivity, as this was outside of the scope of this preliminary study. The second being the use of immunohistochemistry to study the co-localization of connexin 26 with the marker for your cell of interest. This is a qualitative rather than quantitative method and perhaps a method like FISH could also be used in order to give a more accurate measure of expression. The third limitation comes from the use of electrophysiology as a means of measurement. When recording for prolonged periods (>30 min), cells can sometimes be susceptible to run down. To address this, we performed our recordings within a relatively short time period of time. The changes in CO_2_ level that we use are subtle (physiological) and should not therefore cause any cell swelling or lasting distress. However, it can be difficult to routinely get full recovery (Hill et al, 2020). To ensure this partial recovery was not an effect of the initial CO_2_ change, our experiments have been performed in both directions – from 35 mmHg CO_2_ to 55 mmHg CO_2_, and a small number starting in 55 mmHg CO_2_ and changing back to 35 mmHg CO_2_. If raising the CO_2_ was opening for example a KATP channel or resulting in cell swelling and this was responsible for the change in input resistance, then the reverse experiments would not have worked.

***Note:*** Before starting our study, there was already a publication (Vandecasteele et al, 2006) highlighting the expression of connexin 26 in our region of interest using single cell PCR. If this data is not available for your region of your interest then you could consider running single cell PCR, western blot, or preliminary IHC to check for connexin expression profiles before undertaking the study to avoid wasting time.

## Troubleshooting

### Problem

Over the period of recording, the flow of gases may change and hence lead to small changes in pH. It is vital that the experiments are isohydric so that any observed changes can be attributed to the shift in CO_2_ concentration. If expected responses are not observed, the first thing to check is the pH of the solutions.

### Potential Solution

Two strategies are available for this problem, the first is to regularly monitor the pH of the solutions throughout the day to ensure they remain the same. It is also possible to purchase pre-mixed gases for these experiments, although this is a more expensive option and limits the flexibility that you have in testing various different concentrations that is granted by mixing your own gases.

## Resource Availability

### Lead Contact

Further information and requests for resources and reagents should be directed to and will be fulfilled by the Lead Contact, Emily Hill, E.Hill.2@warwick.ac.uk

### Materials Availability

No new mouse lines or reagents were generated for this study

### Data and Code Availability

This study did not generate new code or structural datasets
